# Circumnutation and Growth of Inflorescence Stems of *Arabidopsis thaliana* in Response to Microgravity under Different Photoperiod Conditions

**DOI:** 10.3390/life10030026

**Published:** 2020-03-18

**Authors:** Yuanyuan Wu, Junyan Xie, Lihua Wang, Huiqiong Zheng

**Affiliations:** 1 CAS Center for Excellence in Molecular Plant Sciences, Chinese Academy of Sciences, Shanghai 200032, China; yywu2015@sibs.ac.cn (Y.W.); jyxie@sibs.ac.cn (J.X.); lhwang@sibs.ac.cn (L.W.); 2 University of Chinese Academy of Sciences, Beijing 100049, China

**Keywords:** circumnutation, inflorescence stems, microgravity, photoperiod, *Arabidopsis thaliana*

## Abstract

Circumnutation is a periodic growth movement, which is an important physiological mechanism of plants to adapt to their growth environments. Gravity and photoperiod are two key environmental factors in regulating the circumnutation of plants, but the coordination mechanism between them is still unknown. In this study, the circumnutation of *Arabidopsis thaliana* inflorescence stems was investigated on board the Chinese recoverable satellite SJ-10 and the Chinese spacelab TG-2. Plants were cultivated in a special plant culture chamber under two photoperiod conditions [a long-day (LD) light: dark cycle of 16:8 h, and a short-day (SD) light: dark cycle of 8:16 h]. The plant growth and movements were followed by two charge-coupled device (CCD) cameras. The parameter revealed a daily (24 h) modulation on both TG-2 and SJ-10, under both the LD and the SD conditions. The inhibition of circumnutation was more apparent by microgravity under the SD in comparison with that under the LD condition, suggesting the synergistic effects of the combined microgravity and photoperiod on the circumnutation in space. In addition, an infradian rhythm (ca. 21 days long) on the TG-2 was also observed.

## 1. Introduction

Circumnutation is a revolving movement of rapidly elongating plant organs, such as roots, stems, hypocotyls, branches, flower stalks and tendrils [1,2,3,4]. This oscillating growth pattern depends closely on the growth of the organs as well as the environmental conditions, such as light intensity, photoperiod, mechanical stress and temperature [5,6,7,8]. When growth is interfered with, circumnutation will be reduced or inhibited. For example, when tissues mature and elongation ceases, the circumnutation of these tissues will stop at the same time [9,10]. Arabidopsis seedlings exhibited short-period nutations (20–60 min) at the highest growth rate, while at the low growth rate they exhibited long-period nutations (1–8 h). At a significantly reduced growth rate, the hypocotyls did not circumnutate [11]. This data supports the existence of a threshold on growth rates, below which circumnutation does not occur [12]. However, circumnutation can be blocked without the simultaneous inhibition of growth, suggesting that growth and circumnutation are governed by different mechanisms [13]. The mechanism for circumnutation remains unknown.

Two main models for circumnutation have been discussed. One is an ‘internal oscillator’ model created by Darwin [14], which is connected with the biological clock mechanism that is being investigated extensively [1,13,15,16]. The second approach to circumnutation is biophysical-mathematical, which can be denoted as the ‘gravitropic overshoot model’ [9]. This model was supported by the observations of circumnutation in some agravitropic mutants and plants with abnormal endodermal tissues. The shoot circumnutation of agravitropic mutants, such as *sgr2*, *zig*/*sgr4* and *pgm*, shows a defective or reduced nutational movement [17,18]. In addition, the mutation of *PnSCR* in morning glory (*Pharbitis nil*) results in an abnormal development of the endodermis and also causes a loss of circumnutation and gravitropic response of shoots [17]. However, experiments on STS-2 and -3 [19] and on the International Space Station (ISS) [2,20,21,22] demonstrated that gravity is not required for the maintenance of circumnutation in plants. In these experiments, the oscillations of hypocotyls of sunflowers and the inflorescence (including the primary axillary and lateral inflorescence stems) of Arabidopsis clearly occurred under microgravity. These experiments indicated that circumnutation is probably only a part of the multi-oscillatory system which governs the development and functioning of plants and leads to a variety of oscillatory phenomena [23].

It can be difficult to separate circumnutation from the phototropic, especially gravitropic response interacting with a rhythmic circadian-regulated growth [24,25]. Clear modifications of the circumnutation intensity by photoperiods were reported on ground [6]. However, whether the alteration of photoperiods in the absence of gravity in space will also reveal the same influence on circumnutation is unknown. In this study, we compared the circumnutation of *Arabidopsis thaliana* inflorescence stems under long-day (LD) and a short-day (SD) conditions respectively, under microgravity on board the Chinese space lab TG-2 and the Chinese recoverable satellite SJ-10. The purpose of our study was to examine how photoperiod conditions affect circumnutation under microgravity in space.

## 2. Materials and Methods

### 2.1. Space Experiments

*Arabidopsis thaliana* cv. Columbia plants were grown in the plant culture boxes (PCBs) on board the Chinese spacelab TG-2 and the Chinese recoverable satellite SJ-10, both of which circled the earth about every 90 min providing microgravity conditions under 10^−4^ *g* [26]. The PCBs were developed by SITP (Shanghai Institute of Technical Physics, Chinese Academy of Sciences, Shanghai, China).

**Figure 1:**
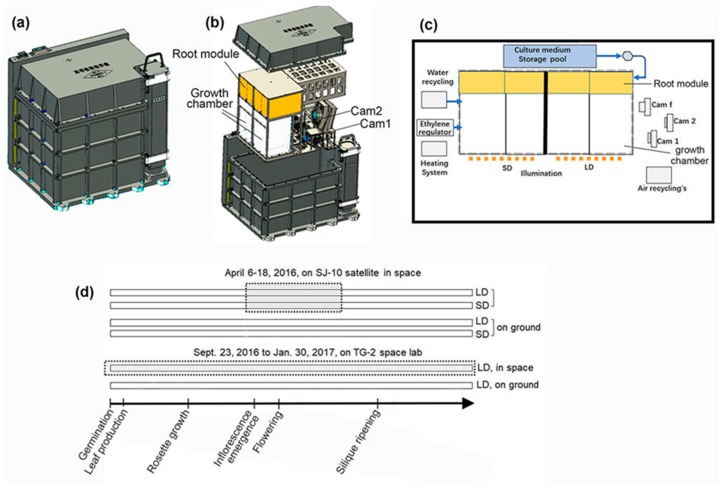
On board model of the plant culture box (PCB). (**a**) The outside view of the PCB (300 × 300 × 400 mm). (**b**) The assembly of the inside view of the PCB with cover. (**c**) Diagram of the composition inside the PCB, showing air, water fluxes, ethylene, nutrient delivery and the image acquirement subsystem. (**d**) Timeline showing the schedule of the experiment and that of the corresponding chronological progression of the principal growth stages in Arabidopsis. Cam, camera; LD, long-day (16 h light/8 h dark) photoperiod; SD, short-day (8 h light/16 h dark) photoperiod; Gray dashed frame, plants grown under microgravity.

On the TG-2, the PCB hardware provided the plant growth habitat for long-term experiments in space (the circumnutation experiment was part of the seed-to-seed experiment) as described by Wang et al. (2018) [27] and Zheng et al. (2019) [28]. Briefly, the PCB provided regulated nutrient delivery, light, air exchange, temperature, and relative humidity to four culture chambers (CCs) (Figure 1a–c). Healthy seeds were selected and surface-sterilized with 75% (v/v) ethanol for 1 min, followed by 2% (v/v) NaClO bleach with 0.01% (v/v) Triton X-100 detergent for 20 min. After five rinses with sterile water, the seeds were dried under a flow bench. Fifteen dry seeds in three rows were set in each root module of the CC containing commercially available vermiculite (Figure 1). The seeds in the CCs were stored drily for about 20 days prior to the launch of the spacelab TG-2 on 15 September 2016. In orbit, initial watering of seeds began about 8 days after the TG-2 launched (23 September 2016). Imaging on the seedlings in the CCs was initiated immediately after the experiment started, with two automated acquisition systems in the PCB at a 2 h interval during the light periods. The plants were grown and imaged for more than 90 days in flight (from seeds to seeds) (Figure 1d). The temperature in the PCB was kept at 22 ± 0.1 °C, and the relative humidity was about 90% during the experiment. The light was turned on 24 h after watering. Illumination was provided by light banks made up of 200 blue/red solid-state light emitting diode (LED) lamps on the long-day (LD, 16 h light/8 h dark) and the short-day (SD, 16 h light/8 h dark) photoperiod, respectively. The photosynthetically active photo flux density produced by LED lamps was 120 μmol m^−2^ s^−1^ at the surface of the root modules. The temperature, humidity and light levels were recorded every 1 min during flight. The PCB was sealed with an air exchange system to provide a slow air exchange (20 mL min^−1^) to the CC with filtered air from the outside system, in which the atmospheric pressure was kept at 1 atmosphere. The data acquired from the space experiment was used to set the ground control experiment. 

The similar PCB hardware used on board the Chinese recoverable satellite SJ-10 provided the plant growth habitat for a 12 days space experiment (the circumnutation experiment was part of plant reproduction experiments) (Figure 1d). Seeds were surface-sterilized as described above and sown on Murashige and Skoog (MS) media supplemented with 0.8% (w/v) agar and 1% (w/v) sucrose. After germination, healthy seedlings were selected and transferred into four root modules (8 seedlings in two rows were set in each root module), which were placed in an incubator (GXZ-1000, Ningbo, China) at Jiuquan Satellite Launch Center for about 18 days. At this age, the plants had formed rosettes and were ready to initiate flowering shoots (corresponding to Arabidopsis growth stage number 3.7) [29], when they were loaded into the PCB less than 24 h prior to lift off on 6 April 2016. In the PCB on the SJ-10 during the 12-d mission, plants were grown and imaged within two CCs. The growth conditions for plants in the PCB on SJ-10 were the same as those on the TG-2, as described above.

### 2.2. Circumnutation Measurement and Data Collection

Two charge-coupled device (CCD) cameras were mounted on the PCB, as described by Wang et al. (2018) [27], allowing the recording of plants in PCB in visible light. Images were sampled at 2 h intervals. Sampled images were stored temporarily on a mass memory unit and were down-linked. 

**Figure 2:**
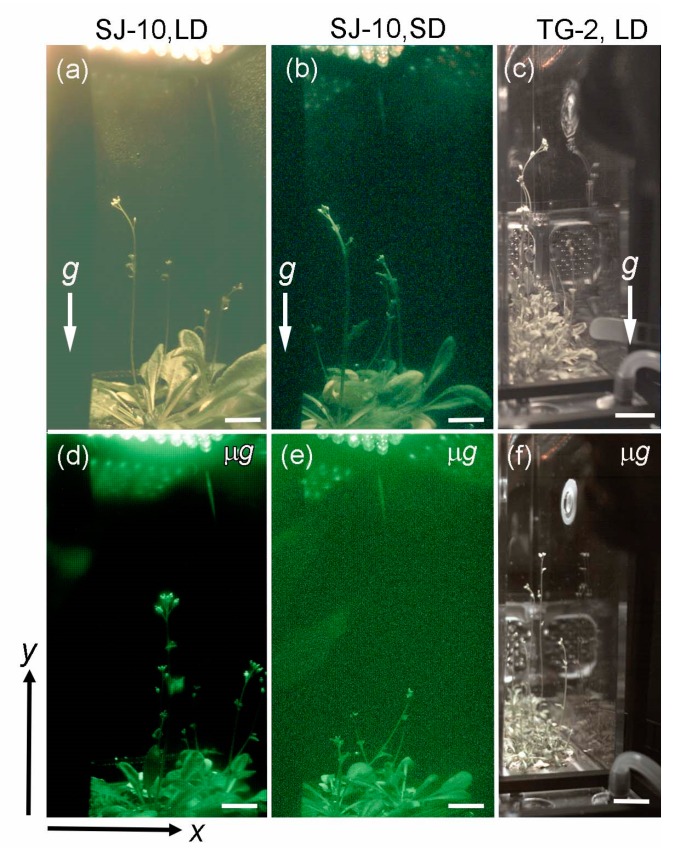
The morphology of space plants and their controls on ground. Examples of images of plants (**a**–**c**) on ground and (**d**,**e**) in space on board the Chinese recoverable satellite SJ-10 and (**f**) the spacelab TG-2 during (**a**,**c**,**d**,**f**) a long-day (LD, light/dark, 16 h:8 h) and (**b**,**e**) a short-day (SD, light/dark, 8 h:16 h) condition, respectively. Positional pixel coordinates were determined for all images analyzed in this study according to the coordinate system in the lower left corner of Figure 2. x-coordinates represent horizontal positions, y-coordinates represent vertical positions. (**a**–**c**) The white arrows denote g, indicating the gravity vector. Bars = 15 mm.

All plant movements are displayed in the x-y plane (the plane defined in Figure 2); because of the 2D limitation of the cameras, only x- and y-coordinates are available. Every image retrieved was interpreted, and the position of parts of shoots were electronically specified in a 2D grid representing the determined x- and y-directions (horizontally and vertically in images, respectively).

### 2.3. Data Analysis

The movements of the inflorescence stems were monitored in space and on ground with CCD cameras using the tracer program. Experimental points were determined at 2 h intervals in the PCB for 12 days on the SJ-10 experiment and for about 90 days on the TG-2 experiment. The images of plants at the stages from just bolting to fully elongated inflorescence shoots were analyzed. The lengths of the shoot circumnutation and heights of plants were estimated with down-linked images using Image J software (http://fiji.sc). Free-running periods and the best-fit curves were estimated from each of five plants by a biomathematical analysis as described in a previous study [1]. To determine whether a periodicity of trajectory length variations existed or not, the data was processed by the Fourier spectral analysis (FFT-NLLS) [30].

## 3. Results

### 3.1. Floral Initiation Rates of Plants in Microgravity on Board the SJ-10 and the TG-2

Because the circumnutation activities of plants were dependent on the developmental stage [18], it was necessary to determine whether or not the floral initiation rates were comparable to those of the ground-based controls before considering the circumnutation of inflorescence shoots in spaceflight. Prior to initiating the SJ-10 satellite experiment, Arabidopsis plants were grown under the LD condition on ground for about 20 days. At this age, the plants had formed rosettes and were ready to initiate the flowering shoots when they were loaded into the PCB less than 24 h prior to lift off. During the mission, the plants developed from the rosette stage at loading to the reproductive stage, with inflorescence shoots elongating under the LD or the SD photoperiod conditions (Figure 2). In this experiment, flowers of the SJ-10 plants were initiated at the same rate as the ground controls whether under the LD (Figure 2a,d) or under the SD conditions (Figure 2b,e). We concluded that there was no significant difference in the floral initiation rates between flight materials on the SJ-10 and ground controls under neither the LD nor the SD conditions, suggesting no discrepancy in the developmental rates between plants grown in microgravity and those grown on ground.

During the 3-months opportunity on the Chinese spacelab TG-2, *Arabidopsis thaliana* completed a seed-to-seed life cycle in microgravity when grown in the PCB habitats under similar physical parameters as in the SJ-10 experiment and also under the LD and the SD conditions, respectively. Plants on the TG-2 space flight germinated and grew as normally as their controls on ground, but the floral initiation of space flight plants under the LD condition was about 20 days later than their controls on ground (Figure 2c,f). Because Arabidopsis is a long-day plant whose flowering under the SD condition is apparently delayed [31], the plants grown under the SD condition in space on the TG-2 failed to initiated floral development during first three months. We only analyzed the modulation of the inflorescence growth and circumnutation of plants under the LD condition in space on the TG-2 and on ground (Figure 2c,f). In addition, to make sure the inflorescence stems of plants grown on the TG-2 and the controls on ground were at the same developmental stage, the bolting time was used as a standard of the developmental stage when comparing the circumnutation of plants in space with their controls on ground.

### 3.2. Circumnutation of Plants under Different Photoperiod Conditions in Response to Microgravity on the SJ-10

**Figure 3:**
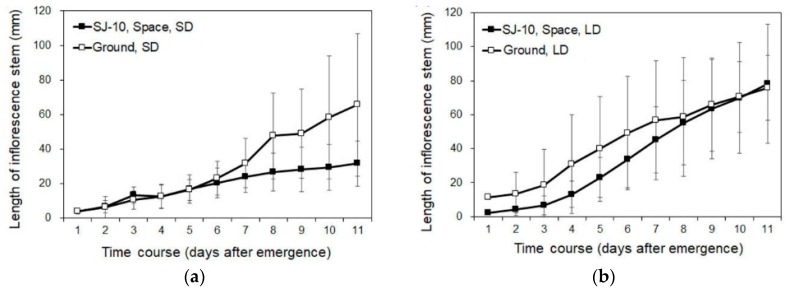
The time course of the elongation growth of inflorescence stems of plants in space and on ground. (**a**,**b**) Plants grown under (**a**) the SD and (**b**) the LD conditions, respectively, in space on board the SJ-10 satellite and on ground. The lengths of the inflorescence stems were measured, and the average lengths of the stems after emergence were calculated as described in the Materials and Methods. Means ± SE (n = 5).

**Figure 4:**
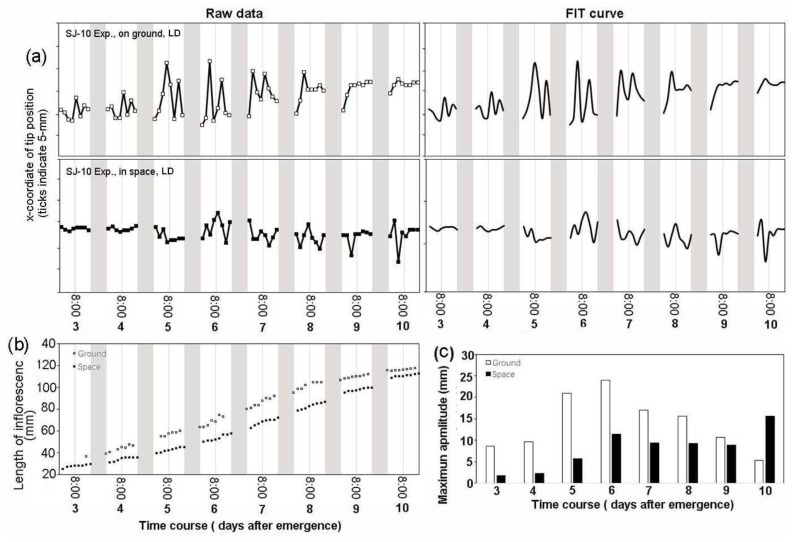
The time course of the circumnutation and growth of inflorescence stems under the long-day (LD) condition in space on board the SJ-10 satellite in comparison with their controls on ground. (**a**) The patterns of the circumnutation amplitude of a representative plant on ground (upper plane: left, raw data; right, the best-fit curves were estimated as described in the Materials and Methods) and in space (lower plane), respectively. We traced five inflorescence stems, and this figure shows one representative example of the plants in space and on ground, respectively. (**b**) The changes in the inflorescence length of the same plants grown in space and on ground, whose circumnutation was described in (**a**). (**c**) Comparison of the average maximum amplitudes of circumnutation of the five inflorescence stems traced every day under the LD condition in space with those of their controls on ground. White areas, light; dark grey panels, dark.

The bolting of Arabidopsis plants under the LD and the SD conditions on the SJ-10 in space was first observed 4 or 5 days after the satellite took off. The average bolting time under the LD condition was 26 days after sowing, and there was no significant difference between the space-grown plants and their ground controls. The elongation growth of inflorescence stems under both the LD and the SD conditions in space was reduced but not significantly in comparison with their ground controls (Figure 3a,b and Figure 4b), suggesting that microgravity did not significantly affect the growth of inflorescence stems. However, the photoperiod could apparently change the growth of inflorescence stems. For example, under the SD condition in space, inflorescence stems exhibited a slow elongation compared to the stems grown under the LD condition whether in space or on ground in comparison with those under the LD condition in space and on ground (Figure 3a,b).

Under the LD condition, circumnutation was not uniform, both in space and on ground (Figure 4a). The amplitude of the circumnutation of the ground control plants was about 5~24 mm during the first 10 days after inflorescence shoot emergence (Figure 4c). For plants grown in space under the LD condition, the amplitude was about 2~15 mm at the same time (Figure 4c). The maximum amplitude of space plants was about 35% smaller than their ground controls (Figure 4c).

**Figure 5:**
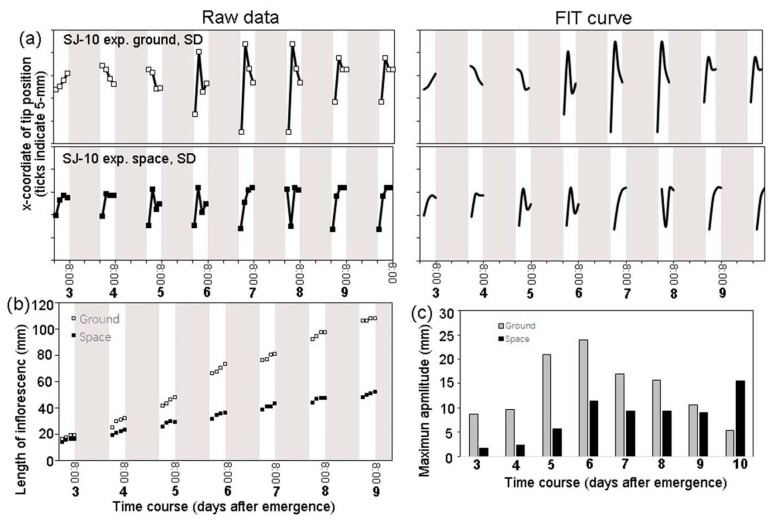
Representative examples for the time course of the circumnutation and growth of the Arabidopsis plant under the short-day (8 h light/16 h dark) condition on board the SJ-10 satellite in space and at 1 g on ground. (**a**). Experimental points (coordinates of the inflorescent shoot apex) were determined at 2 h intervals. The circumnutation amplitudes of plants in space and on ground, respectively. (**b**) The inflorescence lengths of the same plants as described in (**a**). (**c**) A comparison of the maximum amplitudes of circumnutation of plants under the SD condition in space with those of their ground controls. White areas, light; dark grey panels, dark.

Under the SD condition, the circumnutation amplitude of inflorescence shoots on ground was 3~19 mm during 9 days after emergence (Figure 5a,b). For the plants grown under the SD condition in space on the SJ-10, the amplitude was 4~9 mm at the same duration (Figure 5c). The maximum amplitude of space plants under the SD condition was about 52% smaller (Figure 5) than that of their ground controls and about 42% smaller than that of plants under the LD condition in space (Figure 4c). These results indicated that the inhibition of circumnutation was more apparent by microgravity under the SD condition in comparison with that under the LD condition.

### 3.3. Circumnutation of Plants in Long-Term Microgravity Exposure on the TG-2

**Figure 6:**
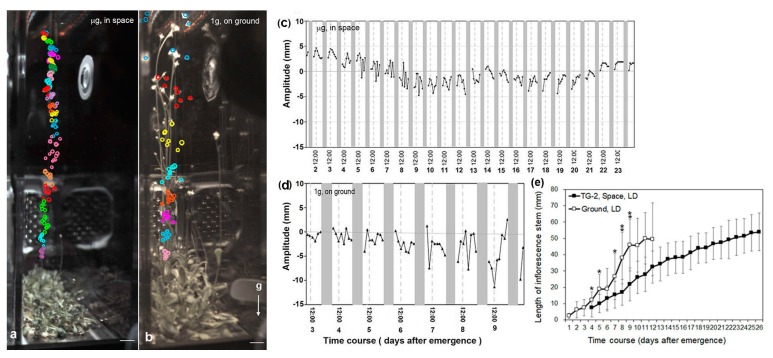
Representative examples for the time course of the circumnutation and growth of the Arabidopsis plant under the LD (8 h light/16 h dark) condition on board the TG-2 in space and on ground. (**a**,**b**) The trajectory of nutation of inflorescence stem apexes (opened circle) of example plants (**a**) in space and (**b**) on ground, respectively. The circles in the same color indicate a daily cycle of circumnutation. Experimental points (coordinates of the inflorescent shoot apex) were determined at 2 h intervals. (**c**,**d**) The circumnutation amplitudes of plants (**c**) in space and (**d**) on ground, respectively. (**e**) The time course of the elongation growth of inflorescence stems of plants grown under the LD condition on board the TG-2 and their controls on ground. Means ± SE (n = 5). * the mean values were significantly different between plants in space and those on ground (* *p* < 0.05; ** *p* < 0.01). Seedlings were grown in the plant culture box on the TG-2 and on ground, respectively, for a whole life cycle (from seeds to seeds). White areas, light; dark grey panels, dark. Bars = 50 mm in (**a**,**b**).

In the TG-2 control experiment, the bolting time of the plants in the PCB on ground was first observed 25 to 26 days after the initial watering, while the average bolting time of plants grown in space on the TG-2 was apparently delayed to 45~50 days. Figure 6 shows a representative example for the circumnutation of the inflorescence stem apexes under the LD condition in space on the TG-2 and their controls on ground, respectively. The movement of the stem apexes in space (Figure 6a) was apparently inhibited in comparison with their controls on ground (Figure 6b). In addition, the elongation growth rate of the inflorescence stems of the TG-2 plants under microgravity also significantly decreased in comparison with their controls on ground (Figure 6e). The average length of the inflorescence stems of ground plants was 128 mm at day 8 after emergence, while plants on the TG-2 in space reached the same length at day 23 after emergence (Figure 6e). The daily growth rates of the inflorescence stems of ground controls slowly increased up to 4 days after emergence and then rapidly increased. For plants under microgravity, the daily growth rates of the inflorescence stems slowly increased up to 15 days after emergence and then decreased. The growth rates under the microgravity condition were 14%–65% lower than those on ground during the first 10 days after emergence (Figure 6e). These results indicated that the differences between the growth rates of the inflorescence stems in space and those on ground were significant. 

The circumnutation of plants under the LD condition on the TG-2 mostly showed smaller daily modulation amplitudes in comparison with the controls on ground (Figure 6a,b). The amplitudes of the circumnutation of plant shoot apexes on ground increased as the growth rates enhanced from 3 days to 10 days after inflorescence stem emergence (Figure 6d,e), but plants grown in space on the TG-2 exhibited a relatively uniform amplitude of circumnutation (Figure 6c,e), which was also observed on the plants grown under both the LD and the SD conditions in space on the SJ-10 (Figure 4a and Figure 5a). These results indicate that the decrease of circumnutation on the inflorescence stem in space is a common physiology response when plants are exposed to microgravity, whether from seeds or from the flowering initiation stage. 

**Figure 7:**
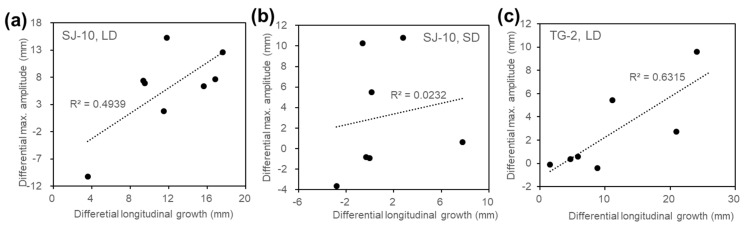
A comparison of the effects of microgravity on the elongation growth and daily maximum amplitude of the circumnutation of inflorescence stems. The differential elongation growth was calculated by subtracting the daily increased lengths of inflorescence stems of plants grown in space from those of their controls on ground, while the differential maximum amplitude was calculated by subtracting the daily maximum amplitude of circumnutation in the inflorescence stems of plants grown in space from that of their controls on ground. The data from plants both on ground and in space during 3 days to 10 days after emergence of inflorescence stems. (**a**,**b**) Plants grown under (**a**) the LD and (**b**) the SD conditions in space on board the SJ-10 and their controls on ground. (**c**) Plants grown under the LD condition in space on board the TG-2 and their controls on ground.

The regression of the data for the maximal amplitudes of inflorescence stems affected by microgravity was associated with the elongation growth under the LD condition both on board the SJ-10 (Figure 7a) and the TG-2 (Figure 7c). In contrast, the correlation coefficient between the alteration of the maximal amplitude and the elongation growth of inflorescence stems under the SD condition on the SJ-10 was only 0.0232 (Figure 7b), about 20-fold smaller than that under the LD condition on the SJ-10 (Figure 7a) and 27-fold smaller than that under the LD condition on the TG-2 (Figure 7c). These results indicate that inhibition of circumnutation of inflorescence stems in space could result from the decrease of their growth only under the LD condition, rather than under the SD condition.

### 3.4. An Infradian Rhythm of Apex Movements of Plants Grown in Space

Interestingly, apart from the daily modulation of the rhythm, the apex movements in plants grown in space on board the SJ-10 and the TG-2 both displayed an infradian rhythm. The Fourier analysis of the time courses under the LD and the SD conditions in space (μg) on the SJ-10 and the TG-2, as well as those on ground (1 g) showed the periodic changes at values that are listed in Table 1. The infradian harmonics were the same in space both on board the SJ-10 and the TG-2 (periodic changes at frequencies of approx. 2, 3 and 5 days). An additional long-period periodic change at frequencies of approx. 21 days was only revealed on board the TG-2.

## 4. Discussion

In this study, we observed that microgravity clearly reduced the amplitudes of plant circumnutation both under the LD and the SD photoperiod conditions. The high-amplitude nutations on earth were correlated with the growth rate of inflorescence stems both under the LD and the SD conditions. This was also observed in space experiments on both the TG-2 and the SJ-10, in which the decrease in the circumnutation amplitudes of inflorescence stems were correlated with the inhibition of the growth rate. In addition, the inflorescence stems of Arabidopsis plants under the LD condition in space both on board the SJ-10 and the TG-2 grew upward toward the light direction in the same way their controls on ground did (Figure 2a,c,d,f). However, the inflorescence stems of plants under the SD condition in space grew in a random fashion; here, the change in plant morphology was recognized as a part of spontaneous growth response called automorphosis [32,33,34]. This was different from their controls under the SD condition on ground, which grew upwards like those under the LD photoperiod (Figure 2b,e). These results indicated that LD photoperiods could overwrite microgravity effects which induce automorphosis under the SD photoperiods. 

The different effects of long-term and short-term microgravity on plant cells have been observed. When exposed to short-term microgravity, plant cells first exhibit an abiotic stress response, such as dramatically increasing the production of ROS and other radicals [35,36], whereas under long-term microgravity exposure plants show a strong capacity to withstand an unfavorable condition by establishing metabolic changes and adaptation to stress [37]. As to planned future long-term manned missions (i.e., Moon base; Mars), it is very important to know the abilities of plants to adapt to the microgravity conditions for 1~2 generations in space flight, where plants are assumed to act as irreplaceable components of Controlled Ecological Life Support Systems (CELSS) in supplying oxygen and food for crew, CO_2_ absorbers and regenerators of water. The growth of inflorescence shoots is a major reproductive trait of Arabidopsis, contributing to seed production and different strategies of plant adaptation to environments. The modulation of the circumnutation speed in Arabidopsis inflorescence stems has been demonstrated to be regulated by a circadian pointing [1], which acts as a central regulator of plant adaptability. Most plant space experiments last less than 18 days, like our experiment on the SJ-10, and only in several space experiments could plants grow from seeds to seeds [38]. Only two space experiments were devoted to a circumnutation study on shoots. In the first spacelab flight, Brown and Chapman (1984) recorded the circumnutation of *Helianthus annuus* hypocotyls in an experiment for about one week. The daily modification of circumnutation under microgravity was observed with a decrease in the amplitude of the movement [19]. Johnsson and colleagues (2009) compared the circumnutation of *Arabidopsis thaliana* stems grown under microgravity with those grown on 0.8 centrifugation on board the International Space Station [2]. These two experiments only analyzed the relevant images over about a one-week duration. Our experiment on board the TG-2 spacelab studied the circumnutation of inflorescence shoots in space over a large time-scale of more than three weeks (CCD cameras were used to follow plant movements from seeds to seeds for more than 90 days). An infradian rhythm was magnified under the LD condition in microgravity on board the TG-2 spacelab with periods of approx. 21 days in comparison with the ground controls. A similar observation in the shoot apical circumnutation under a continuous light, not in an LD photoperiod condition, was reported on ground [6]. The authors suggested that this infradian rhythm of circumnutation could be a response to some environmental periodic changes (the authors assumed periodic gravity changes, such as tidal and lunar, as candidates). Interestingly, the infradian rhythm of circumnutation under the LD photoperiod in our study could only be observed under microgravity but could not be observed in 1 g ground controls. It might be possible that the circumnutation of plants may not only respond to microgravity at a short-time scale by daily modification but also adapt to microgravity at a large-time scale by infradian frequencies connecting with some environmental periodic changes, which could be magnified under microgravity. Many scientists suggested that semilunar and weekly rhythms are caused by moonlight and/or lunar gravity [39,40,41,42,43]. However, this hypothesis requires verification. The origin of the infradian rhythms of circumnutation still remains to be known. Microgravity in space might provide a new opportunity to primarily investigate the influence of lunar gravity on plant growth rhythms on earth.

## 5. Conclusions

In this study, the circumnutation and growth of inflorescence stems of Arabidopsis plants grown on board the SJ-10 satellite and the TG-2 spacelab were analyzed. The findings were as follows. First, LD photoperiods could reverse the microgravity effects which induce automorphosis under the SD photoperiods. Second, the microgravity inhibition of the amplitudes of circumnutation of Arabidopsis inflorescence shoot apexes is related to the photoperiod conditions, and this inhibition was aggravated when plants grew under the SD condition. Third, the inhibition of growth and circumnutation by microgravity is a common physiological response of plants grown in space, whether for the short term (from the rosette stage on the SJ-10) or for the long term (from seeds to seeds on the TG-2). Fourth, the observation of an infradian rhythm in the inflorescence shoot apex movements of plants grown under microgravity in space indicated that the influence of some weak force (i.e., lunar gravity) on plant growth could be masked by the effects of the gravity on earth.

## Figures and Tables

**Table 1:** The periods of the varying trajectory lengths plotted as a function of time in inflorescence stems of *Arabidopsis thaliana* plants grown under LD and SD photoperiod conditions, respectively, under microgravity in space on board the satellite SJ-10 and the spacelab TG-2, in comparison with their controls on ground, were obtained by the Fourier spectral analysis. The most pronounced peak in the spectral density curve is 1, the second one is 2, etc. The periodicities are attributed to periodogram values (P), which refers to the amplitude of the corresponding circumnutation frequency. a.u. is an abbreviation of amplitude unit. Bold, values shared within groups; bold italic, short-period (within 24 h) band; bold upright, long-period (infradian) band. μg, microgravity; 1 g, on ground.

	SJ-10								TG-2			
	LD, 1 g	P(a.u.)	LD, μg	P(a.u.)	SD, 1 g	P(a.u.)	SD, μg	P(a.u.)	LD, 1 g	P(a.u.)	LD, μg	P(a.u.)
1	***24h 00min***	123.0	***20h 48min***	75.2	1d 02h 40min	43.9	3d 08h 00min	32	**1d 09h 36min**	174.8	2d 16h 48min	56.05
2	2d 02h 40min	123.0	**1d 16h 00min**	75.2	**2d 00h 00min**	43.9	**1d 08h 00min**	25.6	**1d 16h 00min**	151.2	**5d 16h 00min**	56.05
3	18h 42min	84.5	**3d 00h 00min**	75.2	**1d 04h 48min**	27.1	**2d 00h 00min**	25.6	1d 05h 20min	151.2	1d 20h 36min	53.9
4	**1d 16h 00min**	84.5	**5d 16h 00min**	64.0	**1d 08h 00min**	8	1d 01h 09min	23.4	***20h 48min***	90.1	**3d 00h 00min**	24.61
5	***20h 48min***	61.2	***24h 00min***	61.7			1d 13h 20min	10.7	***24h 00min***	90.1	4d 14h 24min	24.11
6	1d 02h 17min	60.1	***17h 51min***	59.3			**1d 04h 48min**	9.1	***17h 51min***	66.7	2d 02h 36min	19.49
7	**1d 09h 36min**	50.5	18h 40min	41.9					**3d 00h 00min**	40.0	21d 16h 00min	10.8

## References

[1] Niinuma K., Someya N., Kimura M., Yamaguchi I., Hamamoto H. (2005). Circadian rhythm of circumnutation in inflorescence stems of Arabidopsis. Plant Cell Physiol..

[2] Johnsson A., Solheim B.G.B., Iversen T.H. (2009). Gravity amplifies and microgravity decreases circumnutations in *Arabidopsis thaliana* stems: Results from a space experiment. New Phytol..

[3] Smyth D.R. (2016). Helical growth in plant organs: Mechanisms and significance. Development.

[4] Calvo P., Raja V., Lee D.N. (2017). Guidance of circumnutation of climbing bean stems: An ecological exploration. BioRxiv.

[5] Anderson-Bernadas C., Cornelissen G., Turner C.M., Koukkari W.L. (1997). Rhythmic nature of thigmomorphogenesis and thermal stress of *Phaseolus vulgaris* L. shoots. J. Plant Physiol..

[6] Buda A., Zawadzki T., Krupa M., Stolarz M., Okulski W. (2003). Daily and infradian rhythms of circumnutation intensity in *Helianthus annuus*. Physiol. Plant..

[7] Mugnai S., Azzarello E., Masi E., Pandolfi C., Mancuso S. (2015). Nutation in plants. Rhythms in Plants.

[8] Iida M., Takano T., Matsuura T., Mori I.C., Takagi S. (2018). Circumnutation and distribution of phytohormones in *Vigna angularis* epicotyls. J. Plant Res..

[9] Johnsson A. (1997). Circumnutations: Results from recent experiments on Earth and in space. Planta.

[10] Millet B., Badot P.M., Greppin H., Degli Agosti R., Bonzon M. (1996). The revolving movement mechanism. In Phaseolus: New approaches to old questions. Vistas on Biorhythmicity.

[11] Schuster J., Engelmann W. (1997). Circumnutations of *Arabidopsis thaliana* seedlings. Biol. Rhythm. Res..

[12] Stolarz M., Krol E., Dziubinska H., Zawadzki T. (2008). Complex relationship between growth and circumnutations in *Helianthus annuus* stem. Plant Signal. Behav..

[13] Stolarz M., Dziubinska H. (2017). Spontaneous action potentials and circumnutation in *Helianthus annuus*. Acta Physiol. Pant..

[14] Darwin C.A., Darwin F. (1880). The Power of Movement in Plants.

[15] Whippo C.W., Hangarter R.P. (2009). The “sensational” power of movement in plants: A Darwinian system for studying the evolution of behavior. Am. J. Bot..

[16] Ciszak M., Masi K.E., Baluska F., Mancuso S. (2016). Plant shoots exhibit synchronized oscillatory motions. Commun. Integr. Biol..

[17] Kitazawa D., Hatakeda Y., Kamada M., Fujii N., Miyazawa Y., Hoshino A., Iida S., Fukaki H., Morita M., Tasaka M. (2005). Shoot circumnutation and winding movements require gravisensing cells. Proc. Natl. Acad. Sci. USA.

[18] Hatakeda Y., Kamada M., Goto N., Fukaki H., Tasaka M., Suge H., Takahashi H. (2003). Gravitropic response plays an important role in the nutational movements of the shoots of *Pharbitis nil* and *Arabidopsis thaliana*. Physiol. Plant.

[19] Brown A.H., Chapman D.K. (1984). Circumnutation observed without a significant gravitational force in spaceflight. Science.

[20] Solheim B.G.B., Johnsson A., Iversen T.H. (2009). Ultradian rhythms in *Arabidopsis thaliana* leaves in microgravity. New Phytol..

[21] Correll M.J., Kiss J.Z., Gilroy S., Masson P.K. (2008). Space based research on plant tropisms. Plant Tropisms.

[22] Paul A.L., Amalfitano C.E., Ferl R.J. (2012). Plant growth strategies are remodeled by spaceflight. BMC Plant Biol..

[23] Engelmann W., Johnsson A., Lumsden P.J., Millar A.J. (1998). Rhythms in organ movement. Biological Rhythms and Photoperiodism in Plants.

[24] Jouve L., Greppin H., Degli Agosti R. (1998). *Arabidopsis thaliana* floral stem elongation: Evidence for an endogenous circadian rhythm. Plant Physiol. Biochem..

[25] Dziubinska H., Trebacz K., Zawadzki T. (1999). Circadian growth rhythm of *Helianthus annuus* stem. Folia Histochem. Cytol..

[26] Zhao H., Qiu J., Wang Y., Duan E., Long M. (2019). System design and flight results of China SJ-10 recoverable microgravity experimental satellite. Life Science in Space: Experiments on Board the SJ-10 Recoverable Satellite.

[27] Wang L., Han F., Zheng H.Q. (2018). Photoperiod-controlling guttation and growth of rice seedlings under microgravity on board Chinese spacelab TG-2. Microgravity Sci. Technol..

[28] Zheng H., Wang L., Xie J., Duan E., Long M. (2019). Flowering of Arabidopsis and rice in space. Life Science in Space: Experiments on Board the SJ-10 Recoverable Satellite.

[29] Boyes D.C., Zayed A.M., Ascenzi R., McCaskill A.J., Hoffman N.E., Davis K.R., Görlach J. (2001). Growth stage-based phenotypic analysis of Arabidopsis: A model for high throughput functional genomics in plants. Plant Cell.

[30] Plautz J.D., Straume M., Stanewsky R., Jamison C.F., Brandes C., Dowse H.B., Hall J.C., Kay S.A. (1997). Quantitative analysis of *Drosophila* period gene transcription in living animals. J. Biol. Rhythm..

[31] Yanovsky M.J., Kay S.A. (2003). Living by the calendar: How plants know when to flower. Nat. Rev..

[32] Hoson T., Kamisaka S., Masuda Y., Yamashita M., Buchen B. (1997). Evaluation of the three-dimensional clinostat as a simulator of weightlessness. Planta.

[33] Hoson T., Soga K., Mori R., Saiki M., Wakabayashi K., Kamisakea S., Kamigaichi S., Aizawa S., Yoshizaki I., Mukai C. (1999). Morphogenesis of rice and Arabidopsis seedlings in space. J. Plant Res..

[34] Stanković B., Volkmann D., Sack F.D. (1998). Autotropism, automorphogenesis, and gravity. Physiol. Plant.

[35] Babbick M., Barjaktarović Ž., Hampp R. Alterations in the expression of transcription factors in *Arabidopsis thaliana* cell cultures during sounding rocket μG. Proceedings of the 18th ESA Symposium on European Rocket and Balloon Programmes.

[36] Babbick M., Dijkstra C., Larkin O.J., Anthony P., Davey M.R., Power J.B., Lowe K.C., Cogoli-Greuter M., Hampp R. (2007). Expression of transcription factors after short-term exposure of *Arabidopsis thaliana* cell cultures to hypergravity and simulated microgravity (2-D/3-D clinorotation, magnetic levitation). Adv. Space Res..

[37] Zhang Y., Zheng H.Q. (2015). Changes in plastid and mitochondria protein expression in *Arabidopsis thaliana* callus on board Chinese spacecraft SZ-8. Microgravity Sci. Technol..

[38] Link B.M., Busse J.S., Stankovic B. (2014). Seed-to-seed-to-seed growth and development of Arabidopsis in microgravity. Astrobiology.

[39] Franke H.D. (1985). On a clocklike mechanism timing lunar-rhythmic reproduction in *Typosyllis prolifera* (Polychaeta). J. Comp. Physiol. A.

[40] Spruyt E., Verbelen J.P., De Greef J.A. (1987). Expression of circaseptan and circannual rhythmicity in the imbibition of dry stored bean seeds. Plant Physiol..

[41] Andersson S., Kautsky L., Kalvas A. (1994). Circadian and lunar gamete release in Fucus vesiculosus in the atidal Baltic Sea. Mar. Ecol. Prog. Ser..

[42] Olovnikov A. (2005). Lunasensor, infradian rhythms, telomeres, and the chronomere program of aging. Ann. N. Y. Acad. Sci..

[43] Lüning K., Kadel P., Pang S. (2008). Control of reproduction rhythmicity by environmental and endogenous signals in *Ulva pseudocurvata* (Chlorophyta). J. Phycol..

